# Investigating response time and accuracy in online classifier learning for multimedia publish-subscribe systems

**DOI:** 10.1007/s11042-020-10277-x

**Published:** 2021-01-09

**Authors:** Asra Aslam, Edward Curry

**Affiliations:** Insight Centre for Data Analytics, NUI Galway, Galway, Ireland

**Keywords:** Online training, Internet of Multimedia Things, Event-based systems, Multimedia stream processing, Hyperparameter tuning, Object detection, Smart cities

## Abstract

The enormous growth of multimedia content in the field of the Internet of Things (IoT) leads to the challenge of processing multimedia streams in real-time. Event-based systems are constructed to process event streams. They cannot natively consume multimedia event types produced by the Internet of Multimedia Things (IoMT) generated data to answer multimedia-based user subscriptions. Machine learning-based techniques have enabled rapid progress in solving real-world problems and need to be optimised for the low response time of the multimedia event processing paradigm. In this paper, we describe a classifier construction approach for the training of online classifiers, that can handle dynamic subscriptions with low response time and provide reasonable accuracy for the multimedia event processing. We find that the current object detection methods can be configured dynamically for the construction of classifiers in real-time, by tuning hyperparameters even when training from scratch. Our experiments demonstrate that deep neural network-based object detection models, with hyperparameter tuning, can improve the performance within less training time for the answering of previously unknown user subscriptions. The results from this study show that the proposed online classifier training based model can achieve accuracy of 79.00% with 15-min of training and 84.28% with 1-hour training from scratch on a single GPU for the processing of multimedia events.

## Introduction

The rising interest in multimedia devices with an increase in the number of users is responsible for the evolution of multimedia content in smart environments. This imposes the challenge of processing multimedia events in real-time irrespective of multiple application domains. The Internet of Things (IoT) is designed to support intelligent systems for smart cities, which is responsible for connecting physical things to the Internet. Due to increase in growth of multimedia data in recent years, IoT has to include the notion of multimedia things and thus emerges the concept of the Internet of Multimedia Things (IoMT) in order to facilitate multimedia-based services and applications. Event processing systems are introduced to process data streams for the detection of events within publish/subscribe paradigm, where publish/subscribe is a message-oriented interaction paradigm in which publishers send messages. The consumers express their interest for receiving some useful information [29]. However, event-based systems are more focused on structured (scalar) events [22]. Existing multimedia-based communication technologies have very competitive performance, but all are domain-specific [43, 46, 68, 74]. Monitoring applications related to transport management cannot handle hospital-related events; similarly, ecological surveillance applications will not be able to respond to the events associated with security. To change the application domain, we need to integrate event-based systems with image processing methods each time. Thus existing multimedia applications cannot handle dynamic subscriptions belonging to multiple domains, and we need to move towards generalised multimedia event processing to achieve high accuracy in low response-time.

The goal of generalised multimedia event processing was analysed in our previous work [2], and we reached towards a key open challenge of trained classifiers availability for the processing of multimedia events using neural network-based techniques in real-time. Current online learning approaches make their decisions on the fly [71, 79]. Still, they are only based on concept drift in multimedia streams, and inapplicable for the handling of new/unknown subscriptions belonging to multiple applications of smart cities. Apart from the limitation of availability of pre-trained classifiers, the optimisation techniques in neural network models are based on the trade-off of speed and accuracy [40], which is supposed to be done before the processing of events and cannot be configured at run-time in case of adaptive subscriptions of multiple domains. Therefore, there is the requirement for an online classifier construction-based approach, that can answer known/unknown subscriptions by processing multimedia events with minimal response time and high accuracy.

**Fig. 1:**
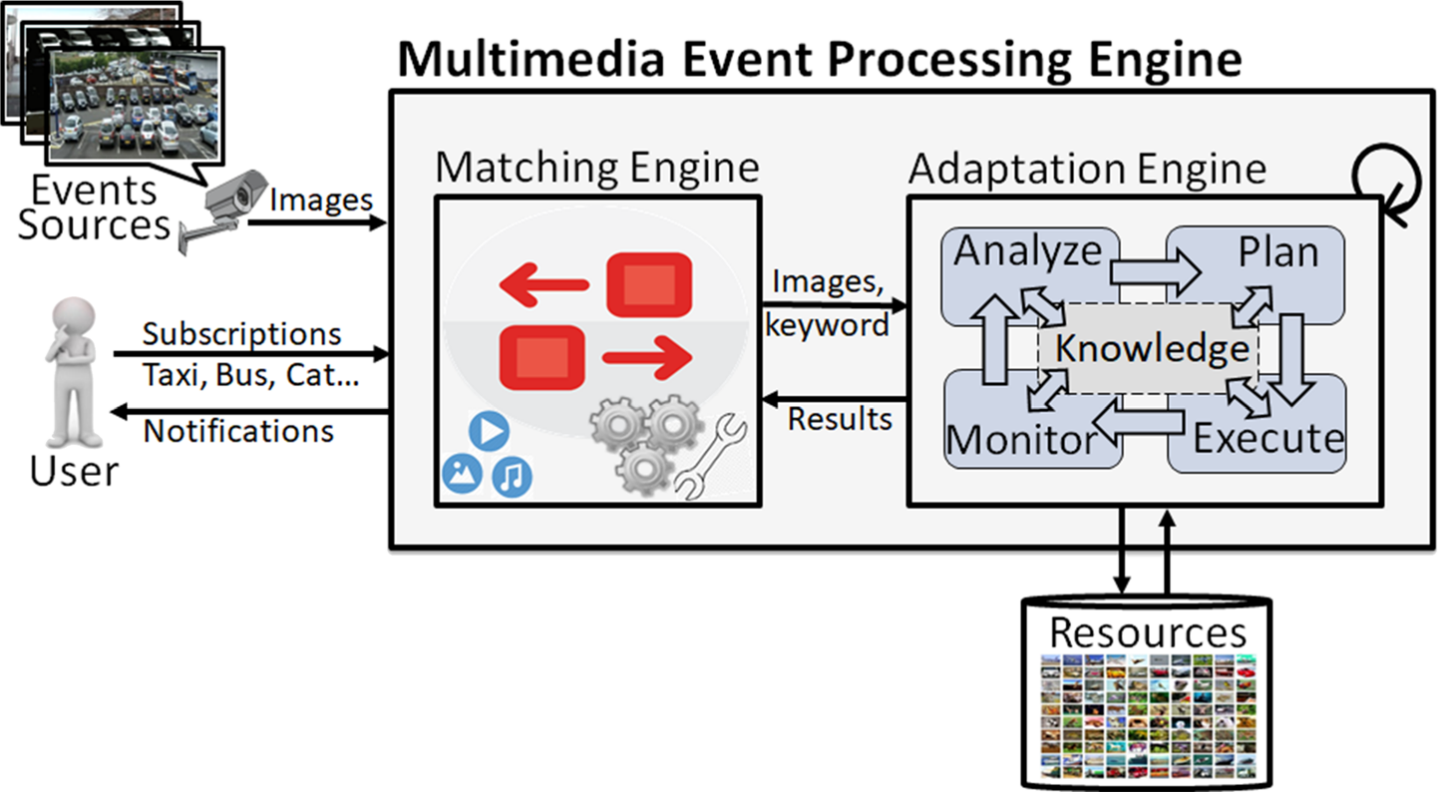
Conceptual architecture for the online adaptive classifier based multimedia event processing

To achieve high-performance multimedia event processing, publish-subscribe based systems are incorporated with online classifier learning-based neural network models specifically for the detection of objects. The multimedia stream processing engine allows users to subscribe to classes belonging to any domain, monitor multimedia events, and process them using an event-based matcher, adaptation model, and a classifier based object detection models (shown in Fig. 1). We optimise the multimedia stream processing model with the help of a self-adaptation model which analyses the accuracy-processing time trade-off of object detection models at run-time and configure it using performance-based strategies on dynamic subscriptions. We leverage hyperparameter tuning based techniques which include the configuration of *learning-rate*, *batch-size*, and *number of epochs* for the optimisation. We consider mainly three strategies: *Minimum Response Time needed while Minimum Accuracy allowed*, *Optimal Response Time needed while Optimal Accuracy allowed*, and *Maximum Response Time allowed while Maximum Accuracy needed*, for the requirement of high performance in multimedia event processing applications.

The contributions of the work can be summarised as follows:

- Formulation of the problem of matching multimedia events with dynamic subscriptions using online classifier training by removing available classifier constraint.
- An adaptive architecture for online classifier construction to minimize the response time and maximize the accuracy.
- Response-Time based strategies with their respective prototypes by tuning hyperparameters for real-time classifier training.
- Evaluation of the proposed approach based on object detection models (YOLO, SSD, and RetinaNet [49, 51, 59]) using multimedia stream events and subscriptions belonging to Pascal VOC and OID datasets [30, 45].

The remainder of the paper is organised as follows. An introduction to the problem, including an outline to motivate the problem, its requirements, definition, and objective function “response-time” is presented in Section–2. A detailed discussion of related work with a brief background description and comparison is presented in Section 3. The proposed “Adaptive Multimedia Event Processing” model, its design, and implementation algorithms are discussed in Section 4. Simulation results and comparisons of the current state of the art object detection models are given in Section 5. Lastly, Section 6 concludes and discusses the implications for future work.

## Problem description

The enormous generation of multimedia data within smart environments with an increasing number of applications requires efficient handling of multimedia-based events. Recently, leveraging the Internet of Things (IoT) for the processing of various large-scale real-time applications is becoming a popular trend in the proliferation of smart cities. Multimedia communication is gradually becoming an essential source of information in multiple domains (like traffic management, security, supervision activities, terrorist attacks, natural hazards). The IoT cannot realise the goal of interconnected objects unless it indeed includes “multimedia” within the processing of information for analysing Internet of Multimedia Things (IoMT) based events. Moreover, in the case of smart cities, subscriptions of users may vary from one domain to another, and require the processing of millions of such dynamic (known/unknown) subscriptions. Thus conventional domain-specific systems become inapplicable and also show variance in performance moving from one application to another.

**Fig. 2:**
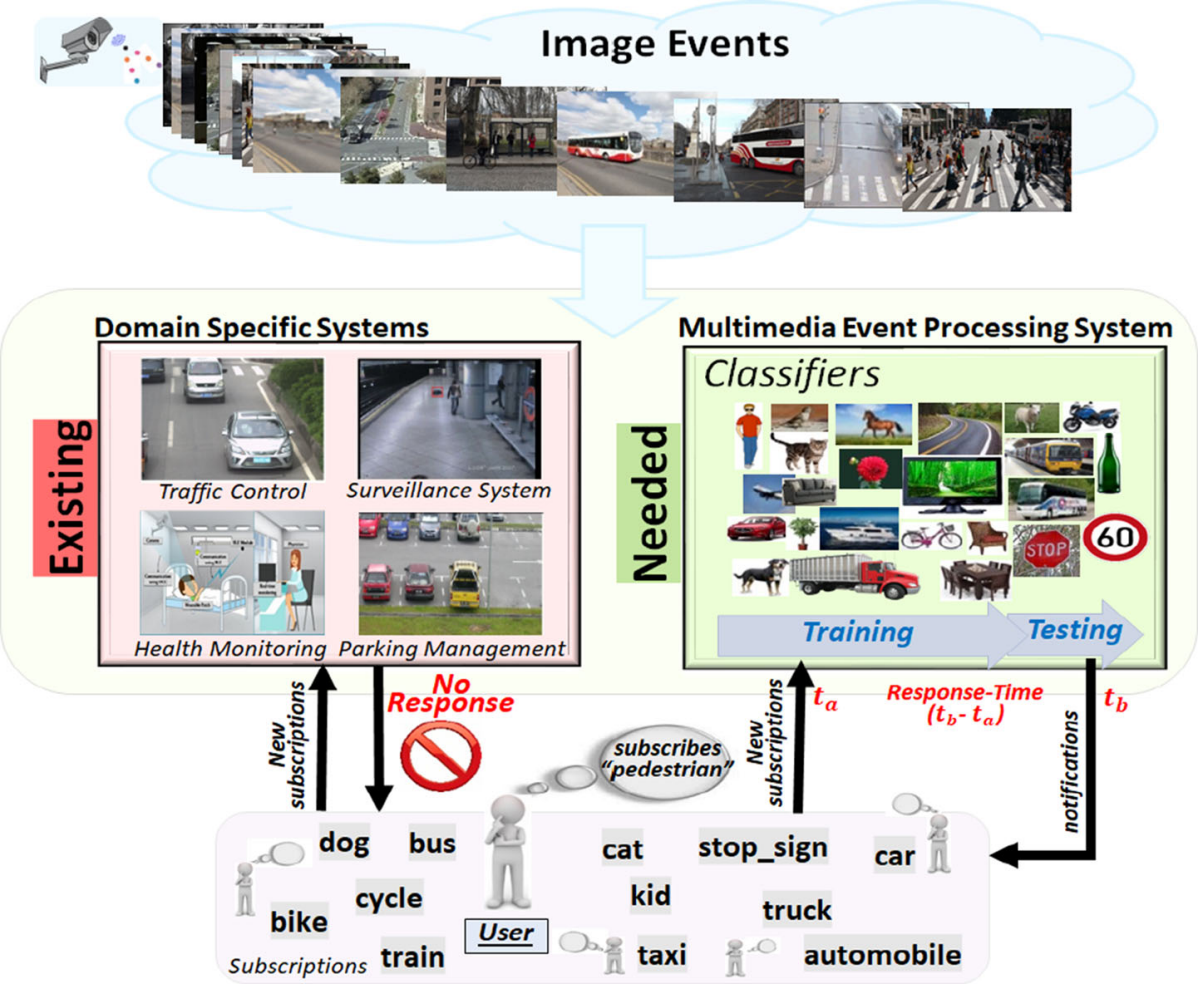
Problem analysis of classifier based generalizable multimedia event processing for multiple types of subscriptions

High performance deep neural network-based technologies with event processing systems could be a possible solution for generalised multimedia event processing. However, such methods impose the constraint of training of classifiers for the matching of multimedia events (shown in Fig. 2). Popular datasets are available only for general classes and not applicable for domain-specific classes. Practically none of the applications are going to require general classes simultaneously, and it is unrealistic to construct a single classifier consisting of all specific classes from all domains. Furthermore, the essential requirement of real-time applications is a high performance, which needs to be fulfilled for its usability. This highlights the need for minimisation of response time while maintaining accuracy, from the perspective of the user.

These drawbacks form the underlying motivation for the presented work, where the proposed online classifier training based multimedia event processing engine utilises the publish-subscribe paradigm and leverages neural network-based object detection methods to meet the requirements of dynamic subscriptions. Here publish/subscribe system facilitates the smooth interactions between subscribers and publishers, and online classifier construction can make the best use of deep neural network-based models using available datasets and adaptive with hyperparameter tuning for specified response-time.

### Motivational scenario

Consider the scenario of object detection for analysing multimedia events as shown in Fig. 2. If a user subscribes for *pedestrian* detection and existing public traffic control management system can recognise only *bus*, *taxi*, *traffic-light*, *etc*. The system may require manual effort for the answering of such unknown subscription “pedestrian”. However, with the provision of online training for the handling of new subscriptions and adaptation of classifiers for reduced response-time, such types of queries can be answered automatically by training a new *pedestrian* classifier while using deep neural network-based models, thereby eliminating the sudden breakdowns that existing systems currently exhibit in such scenarios.

### Requirements

Based on the need for adaptive multimedia event processing within the publish-subscribe paradigm, the following requirements are identified in this work:

- High performance execution method based on the criteria of *maximizing accuracy* and *minimizing response time* of *object detection* models. Here response time includes a short classifier construction time (including data collection & training time) and short testing time. Accuracy is the ratio of correctly predicted observations to the total observations.
- Ability to generalise in order to fulfil the requirement of *data availability* and *maintainability*. Multimedia data consisting of an optimal number of annotated high-quality images, should be available with labelled annotations for the training of the classifiers corresponding to subscriptions belonging to different domains. Here, the ease of transfer from one domain to another with less manual effort represents maintainability.

### Problem statement

The problem can be defined more specifically as “*How can we answer multimedia event* (*S*_*IE*_) *based queries online consisting of completely unseen subscriptions (unbounded vocabulary), using an adaptive classifier construction approach with the tuning of hyper-parameters* (***λ***, $ \boldsymbol {\mathcal {E}} $, ***β***) *while achieving high accuracy and minimising the response time?*”. Before discussion of the proposed approach, we analyse the “response-time” which needs to be minimised for the efficient multimedia event processing.

### Response time

Response time can be defined as the difference between the arrival and notification time of subscription. Suppose a user subscribes at the time “*t*_*a*_” for “pedestrian” and there is no available classifier for the detection of a *pedestrian* in the multimedia event processing system (as shown in Fig. 2). Thus, before the testing (*t*_*t*_) of an image event, the proposed model must need to train (*t*_*tr*_) a classifier first. However, in the worst-case training of classifiers may require data collection (*t*_*dc*_) before the training. Finally, the multimedia event processing system detects events and propagate notifications to the user at time “*t*_*b*_” according to the registered subscription. We can formally define response time (*t*_*r*_) as:

$$ \begin{array}{@{}rcl@{}} t_{r}&=& t_{b} - t_{a}\\ &=& t_{dc} + t_{tr} + t_{t} \end{array} $$

## Related work with background

### Event processing

Event processing systems are designed to process the subscription of a user based on standard languages in response to events [22]. The most popular event-based approaches (CORBA [36], SIENA [16], CEA [4]) rely on producer-consumer paradigm while utilising mediator for providing services and works for supporting application-specific structures. The Common Object Request Broker Architecture (CORBA) uses suppliers, consumers, and single/multiple mediators for providing event-based services. It has a general-purpose event structure consisting of attributes and can be adapted for any domain by employing application-specific attributes. Moreover, it guarantees a soft real-time based quality of services. SIENA [16, 17] is also an event notification service designed for event-based systems to have high expressiveness and scalability. It is extensible,application-specific, and provides best-effort real-time performance. The Cambridge Event Architecture (CEA) also offers a middleware platform that allows producers and consumers to interact using event-based operations. It is flexible, scalable, and also fulfils the requirement of timely response to asynchronous events which is crucial to smart cities-based applications.

There are also many attempts (Hermes [56], ToPSS [14, 21], and Approximate Event Matcher [37]) to bridge the semantic gap between events and language types for the high expressibility of model. Hermes follows the type and attribute-based publish/subscribe model. It introduced the notion of an overlay routing network, where producers and consumers connect to the broker network and individual brokers subsequently route events through the overlay network. The Toronto Publish/Subscribe System (TOPSS) gives an overview of the publish/subscribe paradigm, analysed mobile application requirements, and also implements content-based publish/subscribe paradigm with high throughput. Specifically, it addresses requirements of scalability and ability to support changes, raised by emerging mobile applications. Approximate semantic matching [38] is among one of the current methods, which examines the requirement of event semantic decoupling and investigated the approximate semantic event matching with its consequences. It introduced a semantic event matcher while utilising thesauri-based and distributional semantics-based similarity and relatedness measures.

Similarly, some other approaches (WebSphere MQ [27] and TinyDDS [12]) designed for event-based middlewares supports very few requirements of multimedia event processing systems. WebSphere MQ, currently known as IBM MQ, is an example of MQ brokers, designed for the matching and routing based services along with publish/subscribe messaging services. However, it does not support context-based awareness and composite event handling. TinyDDS is an interoperable and a pluggable publish/subscribe framework which allows Wireless Sensor Networks (WSN) based applications to have control over application and middleware level. It is lightweight, efficient, and provides programming language and protocol interoperability. However, it does not address the heterogeneity and adaptation based requirements.

High-speed nature of event streams with high bandwidth of multimedia data also requires the incorporation of the optimisation techniques in existing event-based systems. However, most common optimisation techniques [5, 42, 63] in these event processing systems are generally based on predicate indexing and network algorithms of matching subscriptions. Predicate indexing algorithms [18, 31, 55, 75] are structured in two phases. The first phase is used to decompose subscriptions into elementary constraints and determine which constraints are satisfied by the notification. In the second phase, the results of the first phase are used to determine the filters in which all constraints match the event. However, the indexing in these approaches are based on the schema of events, and multimedia events are schema-less. Testing network algorithms [1, 15, 32, 35] are based on a pre-processing of the set of subscriptions that builds a data structure composed by nodes representing the constraints in each filter. The structure is traversed in a second phase of the algorithm, by matching the event against each constraint. An event matches a filter when the data structure is entirely traversed by it. Predicate based grouping in these algorithms is based on attribute values and thus can fail to support multimedia event processing.

It can be observed that all of these existing publish-subscribe based event processing systems are only focused on structured (scalar) events for the processing of subscriptions of a user, with no provision of handling and optimising events consisting of multimedia data.

### Multimedia stream processing

Multiple applications are designed for different roles like traffic management, parking, surveillance, health monitoring, and various supervision activities in smart cities. These numerous applications are highly efficient in processing multimedia (unstructured) systems events but applicable for only specific domains. A real-time traffic sign recognition scheme [46] is proposed to assist driving using smart-phones. The proposed model includes five different stages: (1) video/frames capturing using a smart-phone, Notebook, and other computer devices, (2) then preprocessing stage improves the image quality and perform normalisation operations, (3) traffic sign detection step monitors frames to detect the region of traffic signs if they exist, (4) extracts the detected sign, and (5) finally a model recognises the character/icon. Their experiments proved that the model could achieve accuracy up to 98% while having a recognition speed of 0.085 seconds per frame. However, this model is applicable only for *traffic-signs* detection, and even those signs assumed to have either rectangular or circular shape. Similarly, another research [43] focused on the problem of traffic light switching according to traffic congestion on the road. This system consists of 4 video cameras on the traffic junctions; then it takes one image of the empty lane as a baseline to compute the density of vehicles on the road. Then it keeps monitoring the density of vehicles present every second, for all the lanes where light is red. Then the time for the green light signal is calculated using the number of vehicles that can pass in one second using the records of density. Results for traffic light switching show that the model can improve the time for passing the vehicles up to 35% approximately. Another work [26] intends to provide the Internet of Vehicles (IoV) based traffic management solution. Proposed IoV focused on communications of four types: communication between the vehicles and the vehicle owners, communication between vehicles, communication between vehicles and a centralised server and communication between the server and third parties (emergency response, pollution control, police patrol). Advantages of IoV include *traffic control*, *human proximity detection*, *theft avoidance*, *accident avoidance*, *emergency response*, and *vehicles-autonomous*. Its identified drawback is related to *security* and *failure of networks*.

Similarly, an example of smart surveillance systems for airport security is considered in [68]. This IBM S3, smart surveillance system, has two key components, namely, Smart Surveillance Engine (SSE) and Middleware for Large Scale Surveillance (MILS). SSE is responsible for performing event detection and supports video/image analysis. MILS supports the indexing and retrieval of spatio-temporal event meta-data. The example shown is the integration of many technologies like license plate recognition, behaviour analysis, face detection, and badge reading. However, the proposed system satisfies the requirements of *openness* and *extensibility*. However, the S3 framework has its own *airport system data model*, *user data model*, and *event data model*. Boll et al. [11] focused on the problem of analysing multimedia events for health monitoring. The proposed logical device layer-based architecture maps data from one or multiple (logical) devices into primary health features. Presently, the mapping for primary health features is canonical, i.e. scale directly delivers the values of body weight and fat. However, it could be extended for complex event processing like identification of “20 minutes cycling to work” using the time of day, GPS track, step counts, and past observations. An IoT based agricultural production system also proposed to analyse crop environments and improve the efficiency of decision making [47]. The designed system forecast agricultural production by monitoring crop growth periodically using IoT sensors. The system architecture can be divided into parts: relation analysis, statistical prediction, and IoT service. In statistical prediction, the production amount gets computed by estimating cultivated area and yield functions. It utilises text mining technology for relation analysis while analysing correlations of the agriculture-related text and locational conditions, selection, and replacement of crops. IoT service serves as an invaluable component that continuously monitors equipment and reports in real-time about the environment’s conditions. Lastly, the design is implemented along with a GUI for visualisation.

It can be concluded existing multimedia based real-time systems possess high efficiency, but most of them are domain-specific. Moreover, low expressiveness for user query and a lack of provision for the publish-subscribe paradigm [29] also limits the application of these systems in multiple scenarios of smart cities. Thus, merging event-based systems [22] with image processing systems is an essential requirement of current multimedia stream processing, which also limits the performance and requires high setup cost. In our previous work [2, 3], we proposed an approach for generalised multimedia event processing using deep neural networks for the IoMT based data and also realised the requirement of pre-trained classifiers for the handling of multiple domain-based subscriptions.

### Online learning of classifiers

Online learning-based approaches make their decisions on the fly and applicable for situations in which data changes frequently [67]. Thus such machine learning-based approaches could prove to be useful for the adaptation among classifiers. These algorithms are scalable and data-efficient that learn to update models from data streams sequentially, and no longer require data which has been consumed [39, 73]. Data streams frequently experience “concept drift” as a result of changes in the underlying concepts. Online learning-based approaches are adaptable and can easily handle concept drifting of data streams, which makes this methodology crucial for streaming analytics [79].

Most of the existing techniques of online learning are based on semi-supervised or active learning of classifiers. Active learning is one of the techniques that allow the machine learning methods to select a subset of the unlabeled data from the data distribution to be labelled [19]. Uncertainty sampling [48], Query-by-Committee (QBC) [66] and Estimation of error reduction [64] are the most popular methods to perform active learning. Although active learning is an enhancement over conventional inductive learning; the approach requires the construction of an exhaustive labelled dataset which is laborious and challenging [54]. Semi-Supervised learning is also another step towards online learning which requires a small amount of labelled data as compared to unlabeled data. These algorithms [24, 53] work on the hypothesis that the labels generated by the base learner with high confidence can be added to the training dataset, and able to improve the accuracy. Although the accuracy of such existing methods is relatively high with high speed of stream processing, given that these methods are semi-supervised, they cannot handle multimedia streams online.

Existing adaptive classifier based machine learning techniques [23, 71, 78, 79] in this category are also designed with the aim of the evolution of classifiers with drift in the concept of data streams. An ensemble of classifiers is based on combining the results of individual classifiers and producing more accurate results for dynamic data streams, thus suitable for online learning [33]. Wang et al. [71] proposed a framework for concept drifting of data streams using weighted classifier ensembles. However, the identification of *concept drift* in these dynamic approaches is mainly focused on the processing of structured data streams and cannot accommodate multimedia data streams. Moreover, it has been investigated that the dynamic ensemble selection scheme performs better than static ensemble selection in some cases [44]. Other recent adaptive classifier based techniques [23, 78] are efficient but applicable only for particular domains. Furthermore, taking the decision only by analysing multimedia streams and adapting classifiers is not sufficient for the answering of unknown subscriptions belonging to multiple domains of smart cities, as it may require the publish-subscribe paradigm for the purpose of communication and processing user queries.

Online learning can be directly applied to deep neural networks, but also suffers from convergence issues and forgetting previously learned data [65]. Although online learning-based approaches remove the constraint of availability of classifiers, most of them are solely based on concept drift in multimedia streams and thus become inapplicable for handling dynamic subscriptions. Moreover, handling the challenge of changing/inconsistent interest of the user, and adapting classifiers accordingly, need to be investigated for the generalized framework of multiple domain-based streams [77].

### Self-tuning of classifiers

Hyperparameters are configuration parameters of the model that cannot be trained directly from the training data, and often specified by practitioners after resort to experimentations. Examples of hyperparameters may include learning rate, number of epochs, batch size, number of hidden layers, architecture, activations functions, etc., and choosing the right set of these values is typically known as *Hyperparameter tuning*. Since the choice of hyperparameter values greatly affects the performance of resulting classifiers, various automatic selection methods were proposed in the literature [52]. The most common algorithms for the selection of hyperparameters are ranging from Grid Search, Random Search, Bayesian Optimization, Sequential Model-Based Optimization to Tree-structured Parzen Estimators (TPE) [10]. Grid search is the simplest method of hyperparameter tuning. It is a brute force method that trains the model for all combinations of parameters specified in a grid and selects hyperparameters after evaluating each model. Since grid search suffers from having high dimensional space, it is computationally very expensive. Random search is different from a grid search as it assumes that not all hyperparameters are equally important. In this method, we provide the statistical distribution for each hyperparameter from which values may randomly sample. We may also define the total number of iterations, and the hyperparameter values of the model will be set and evaluated for each iteration from a specified probability distribution. As compared to the grid search, the random search has much improved exploratory power [7]. Bayesian approaches keep track of the previous iteration results to improve the sampling method for the next experiment [69]. There are two main decisions that we need to make for Bayesian optimization: (1) Selecting a prior over functions to express assumptions about the function being optimized (for instance Gaussian Process prior) (2) Choosing an acquisition function to determine the next point to evaluate. Sequential model-based optimization (SMBO) algorithms formalize Bayesian optimization [10]. It iterates between fitting models sequentially while trying each time better hyperparameters using Bayesian reasoning and updating the probabilistic model.

Many variants of SMBO algorithms exist which differ only in the surrogate model, where the surrogate is the model used for approximating the object function. TPE builds a surrogate model by applying Bayes’s rule [9]. This method is restricted only to tree-structured configuration spaces, i.e. leaf variables only make sense when node variables take particular values. TPE first samples the hyperparameter search space by random search, then it divides the output scores into two groups. The first group consists of best scores and the second group contains the rest of the observations by assuming *y*^∗^ as the splitting value for the two groups. Then the two densities *l*(*x*) and *g*(*x*) are modelled using Parzen Estimators, where *l*(*x*) and *g*(*x*) are averages computed from kernels centred on existing data points. The TPE algorithm defines likelihood probability as *p*(*x*|*y*) = *l*(*x*) if *y* < *y*^∗^ or *p*(*x*|*y*) = *g*(*x*) if *y* ≥ *y*^∗^. The model evaluates the sample hyperparameters according to *l*(*x*)/*g*(*x*), updates observation list, and iterates over a fixed number of trials. The major advantage of the TPE is that it allows a vast domain for hyperparameter search space. These baseline methods are further integrated into open source softwares for the automatic selection of algorithms and hyperparameter values. Auto-WEKA [70] is one of the most popular works towards analyzing machine learning algorithms automatically and setting appropriate hyperparameters in-order to enhance performance. Similarly hyperopt-sklearn is another available software that mainly includes random search and TPE for the automatic selection [8].

We can conclude that these existing methods are automatic, but most of them are focused only on the accuracy and generalization ability of classifiers, or on the computation cost consisting of testing time while excluding the training time of neural-network-based models. Thus existing adaptation tools designed for tuning of hyperparameters need to be further investigated for minimizing the overall response time, including both testing and training time. Analysis of current approaches using requirements (presented in Section 2.2) is shown in Table 1 for the comparison of work related with the proposed system.

**Table 1:** Comparison of related-work with requirements

Approach	Requirements			
	High-Performance		Ability to	
	Execution Method		Generalize	
	Maximising Accuracy	Minimising Response Time	Data Availability	Maintainability
Event Processing [4, 12, 16, 21, 27, 36, 37, 56]	Accurate	Low Response Time	N.A	N.A
Multimedia Stream Processing [11, 26, 43, 46, 47, 68]	High Accuracy	Low Response Time	N.A	N.A
Online Learning of Classifiers [23, 71, 78, 79]	Average Accuracy	N.E	N.A	High Maintainability
Self-Tuning of Classifiers [7, 9, 10, 69, 70]	High Accuracy	N.E	N.A	N.A
Our Adaptive Multimedia Event Processing Model	Average Accuracy	Minimum Response-Time	Expandable	Adaptable

N.E: Not Evaluated, N.A: Not Addressed

## Multimedia event processing

In this section, we first discuss the proposed approach of the adaptation engine in Section 4.1 with its designing and implementation algorithms in the proceeding Sections 4.2 and 4.3.

### Approach

**Fig. 3:**
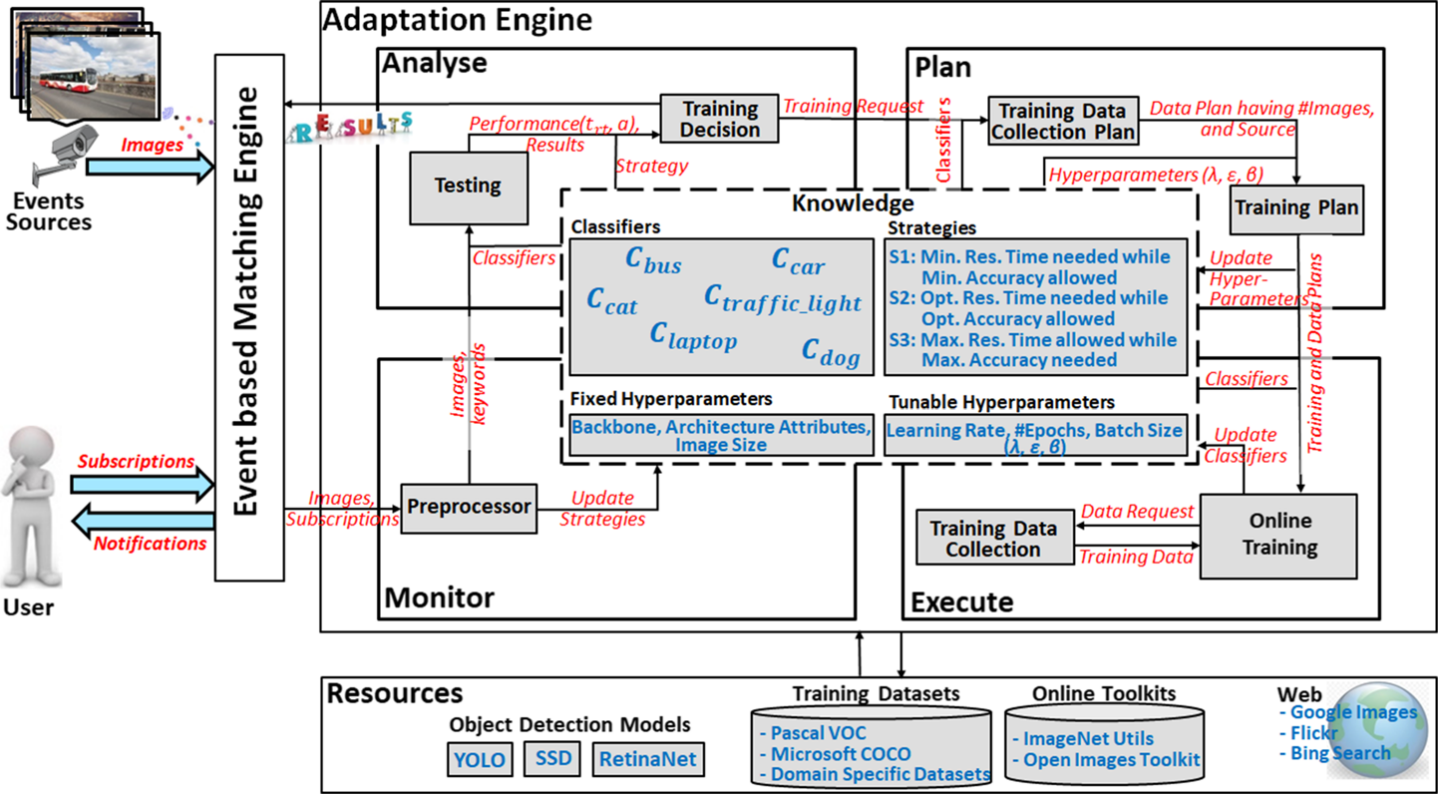
Adaptation model for multimedia event processing

The proposed online classifier learning based multimedia event processing model utilises publish-subscribe paradigm and leverages neural network-based object detection methods to meet the requirements of dynamic subscriptions. Publish/Subscribe system facilitates the smooth interactions between subscribers and publishers sending multimedia events. The adaptive multimedia event processing engine allows users to subscribe to classes belonging to any domain, monitor multimedia events, and process them using an event-based matching engine, adaptation model, and external resources (shown in Fig. 3). The event-based matching engine is responsible for the detection of conditions which hold in image events according to the user query. Deep convolutional networks based object detection models are included for the processing of multimedia events with high performance, and currently being placed in resources that can be changed/adapted on need by administrator only. Adaptation model has been incorporated for online configuration of classifiers, so that the system can adapt and train new classifiers based on suggested strategies, on the arrival of unknown/new subscriptions. Proposed adaptation model derives the best configuration for the considered strategy by analysing the response time-accuracy trade-off of image processing models (presently object detection). It is important to note that we are using the term “adaptation” for the tuning of hyperparameters based on strategies categorised by response-time. However, other types of adaptations could be incorporated in future for the enhanced efficiency of the proposed architecture.

Adaptation model has been designed using IBM MAPE-K architecture [20] having *Monitor*, *Analyse*, *Plan*, and *Execute* phase, a shared *Knowledge Base*, and managed *resources*, shown in Fig. 3. The monitoring function is responsible for receiving subscriptions (in the form of keywords), image events, and any other specified requirements. The analyse phase process images and take decisions of training or testing based on the performance of available classifiers. The planning phase configures tunable parameters to start training based on a decision of construction of classifiers. The execution phase initiates the training of the classifier. Meanwhile, almost all of the phases interact with the knowledge base to access configurations, policies, strategies etc. The last layer contains managed resources (hardware or software) that assist the adaptation engine and presently include training database and neural-network-based models.

### Design and implementation

The proposed approach for the adaptive multimedia event processing (shown in Fig. 3) illustrates the online learning of classifiers on demand along with hyperparameter tuning based optimisation and addresses the monitoring, analysing, planning, and execution phase using knowledge base and resources as follows:

#### Monitoring

Firstly the monitor phase is responsible for receiving subscriptions (in the form of keywords like *pedestrian*, *bus*, *cat* etc.), image events, and any other specific requirement (for instance *strategies*) suggested by the user. Choice of strategies may vary with applications; presently we consider mainly three strategies: “Minimum Response Time needed while Minimum Accuracy allowed”, “Optimal Response Time needed while Optimal Accuracy allowed”, and “Maximum Response Time allowed while Maximum Accuracy needed”.

#### Pre-Processing

It is the responsibility of the preprocessor to update strategies if instructed by the user and then communicates with the analysis phase for providing subscriptions (keywords) and images.

#### Analyse

The analyse function is designed to evaluate image events and analyse the performance to determine if some changes (specifically training) need to be done. It mainly includes testing and training decision phases, utilise the knowledge base for existing classifiers and requested strategy.

*Testing:* The “testing module” processes image events using classifiers belonging to keywords and object detection models with testing configuration parameters from the shared knowledge base and resources.
*Training Decision:* The training decision phase utilises the results generated by the testing phase and strategies from the knowledge base to start the training or continue with testing. It analyses the performance (response-time and accuracy) of the testing module and requests for a training plan accordingly. If response-time (*t*_*rt*_) and accuracy (*a*) satisfy the requirements of subscribers, the analyse module communicates results to the event matching engine.

#### Plan

This phase creates or selects a procedure for the training data collection and generates the training plans using classifiers and hyperparameters present in the knowledge base.

*Training Data Collection Plan:* The data collection plan gets initialised from the training request of the analyse phase, then it considers available classifiers, and training data present in resources, to make the data collection plan. It may also consider the collection of training data from an external source like automatic data collection tools like OIDv4_ToolKit [45] in the present case. ImageNet-Utils [28] is also another typical example of an online data collection tool, which includes more than 1000 categories for user subscriptions. Other than these resources, classifiers for such unknown subscriptions could also be constructed using search engines like *Google Images*, *Flickr*, *Bing Image Search API*, *etc.*, and automatically downloading images using class names.
*Training Plan:* The training plan receives the data plan with details of sources and the number of images, and fetches existing hyperparameters from the knowledge base. It decides the training time by considering the requested strategy and data plan and estimates hyperparameters to give the best performance in the limited response time. Lastly, the training plan updates the hyperparameters (like Learning Rate, Number of Epochs, Batch Size) present in the knowledge base and invokes the *training* module.

#### Execute

Execution function provides the environment of online training of classifiers and performs the required changes to update the classifiers necessary for the adaptation of the system.

*Online Training:* This module mainly performs the training of classifiers for unknown subscriptions using the training plan generated in the previous phase. It may also collect the classifier (if it exists) from the knowledge base and train further following the training plan. Also, training can take place in parallel to testing in the distributed systems. Finally, classifiers get updated in the knowledge base after reaching the training time decided by the planning phase according to the requested strategies.
*Training Data Collection:* The training module could also instantiate the data collection function for the collection of training data from an external source (presently OIDv4_ToolKit [45]). It may also consider details like the number of images, size, quality, etc., from previously generated data collection plans. It provides the requested data within the specified time directed by the online training module. Moreover, it updates resources with collected data for the processing of the same subscription in the future.

#### Knowledge

It represents a shared knowledge accessible from all phases in different situations and may consist of the following components:

- Classifiers: Testing and training module interacts with classifiers using subscriptions and updates them on need. In the present scenario, classifiers get trained online for the new/unknown subscriptions while collecting training data either online or offline. For instance, for the classes present in Pascal VOC, Microsoft COCO, ImageNet object detection datasets, etc., the model directly collects data from resources offline for the training of classifiers. However, in case of a completely new subscription, the model chooses the possibility of collecting training data online either from existing online data collection toolkits or from web sources.
- Configuration Parameters (Fixed and Tunable): Classifier configuration may vary with the adaptation of tunable hyperparameters. In the current implementation, we fixed the architecture, image size, backbone, etc., and tuned the batch size, learning rate, and the number of training epochs. We use the Tree-structured Parzen Estimators (TPE) method for the tuning of hyperparameters [10] which is the most recent and fixes the limitations of conventional optimisation techniques (please refer to Section 3.4).
  Hyperparameters are those parameters of the model whose values get set before the training starts. Setting hyperparameters is critical as they directly affect the behaviour of the training and significantly improves the performance (response-time and accuracy) of the model. On the other hand, there is very little research related to ways of choosing hyperparameters for tuning [57]. However, hyperparameters generally classify as *optimiser* hyperparameters and *model-specific* hyperparameters. The optimiser hyperparameters are more focused on the optimisation of the training in terms of efficiency and accuracy. The model-specific hyperparameters are related to the design of the model. A typical set of optimisation hyperparameters for neural networks based models includes learning rate, batch size, and the number of epochs, which we also consider in our adaptation model for the tuning. The learning rate is the most important hyperparameter that has to be tuned [6]. If the learning rate is too low, then it will increase the response time, or if it is too large, the model will never converge. Batch size is also responsible for speed and number of iterations in training. Moreover, larger batch size consumes more memory while smaller batch-size induces noise. Choosing the batch size determines the number of iterations, and the length of the epoch depends on the number of iterations. Thus the batch size and the number of epochs are directly related to the training time of the model, and we must need to consider such hyperparameters for tuning.
  On the other side, attributes that control the architecture of the neural network like the number of layers, activation function, backbone, also fall under the category of hyperparameters, but these parameters are model-specific. We are keeping these elements of specific architectures of each model fixed. However, we are changing the full architectures by changing the object detection models (YOLO, SSD, and RetinaNet) and discussed evaluations in Section 5.3. Specifically, the SSD model uses *VGG-16* as the backbone and adds 6 convolutional layers while using Softmax as an activation function [51]. RetinaNet model comprises *ResNet-FPN* backbone, a classification subnet, and a box regression subnet, where both classification and box subnet consist of 5 convolutional layers and ReLU based activations [49]. YOLO uses its backbone *darknet* with 24 convolutional followed by 2 fully connected layers and uses linear activation function [59]. The recommended image size for the SSD model is 300 × 300, RetinaNet model is 800 × 1333, and YOLO is 448 × 448. If we modify the image-size, then that would considerably change the testing time with less change in training time. However, this would compromise the accuracy, and we will eventually need more images during training to reach the same accuracy. This will result in more training time and more data collection time in the worst case. Similarly, tuning individually, these parameters could change the specific architectures of object detection models and may enhance the speed of training but not significantly. However, there exist comprehensive reviews [40, 41, 76] that are changing feature extractors (backbones), activation functions, proposals, layers, image size etc. of these models in the field of object detection, but ideal architecture with its parameters is inconclusive to date. Our model is also extensible for the adaptation within the object detection models by using such existing recommendations and incorporating them in the knowledge base and planning phase of our model. Although, the complexity of our proposed adaptation model will increase with an increase in the number of dimensions, and we will need to give priorities only to a few *model-specific* parameters or only to *optimisation* parameters in the end.
- Strategies: It is important to note here strategies refer to user requirements for performance. Suppose a user permits low accuracy results but in minimum possible time then this strategy can be attributed to as “Minimum Response Time needed while Minimum Accuracy allowed”; Conversely if a user necessitates high accuracy results with no restriction on response time then one can specify strategy “Maximum Response Time allowed while Maximum Accuracy needed”. Similarly, any other choice of response time that supports accuracy between low to high may fall into the category “Optimal Response Time needed while Optimal Accuracy allowed”.

#### Resources

This component consists of existing image processing models and training datasets. For the demonstration of the proposed model, we are using YOLO, SSD, and RetinaNet for object detection [49, 51, 59]; and Pascal VOC and OID [30, 45] with its online toolkit^1^ for training datasets. However, resources may include toolkits like ImageNet_Utils^2^ or web sources like Bing Scrapper^3^, Google Images Downloader^4^, Flickr_Photos^5^, etc.

Moreover, we could also incorporate other recent object detection models in the future. Presently we analyse the most recent object detection models (shown in Table 2): *Faster-RCNN* [62], *Region-based Fully Convolutional Networks (R-FCN)* [25], *Single Shot MultiBox Detectors (SSD)* [51], *Deconvolutional Single Shot Detectors (DSSD)* [34],*You only look once(YOLO)* [59], and *RetinaNet* [49], based on their performance. Here, we focus on the testing time of these models, as after the training of any unknown subscription, the response-time of our model will depend only on the testing-time. Thus we chose the YOLOv3 and SSD300 due to their lowest testing time. Moreover, RetinaNet is the most recent among these models, and it is getting popular due to its highest accuracy (to date), we consider it in our experiments. Nonetheless, we could include other object detectors depending on the requested strategies. Please note the average precision and inference time of object detection models are the best results reported by these models, thus may differ in future with an increase in resources.

**Table 2:** Performance of existing object detection models

Current Object Detection Models	Performance	
	Precision*	Testing Time (in ms)
Faster R-CNN [62]	0.27	420.00
R-FCN [25]	0.32	170.00
SSD300 [51]	0.25	21.74
SSD512 [51]	0.29	52.63
DSSD321 [34]	0.28	105.26
DSSD513 [34]	0.33	181.82
YOLOv2 [60]	0.22	25.00
YOLOv3 [61]	0.28	22.00
RetinaNet [49]	0.41	198.00

^*^ average precision on coco test-dev @IOU[0.5, 0.95] [50]

### Adaptive multimedia event processing algorithms

The implementation procedures for online multimedia stream processing engine with adaptation are shown in Algorithms 1 and 2, where *S*_*s*_ represents sets of Subscriptions, *k*: keywords, *s*: subscribers, *S*_*IE*_: stream of image events, *M*: object detection model, ***λ***: domain for learning rate, $\boldsymbol {\mathcal {E}}$: domain for number of epochs, ***β***: domain for batch size, *C*_*k*_: classifier for keyword k, and *St*: strategies respectively.

Algorithm 1 gets instantiated with subscriptions consisting of keywords subscribed by multiple subscribers. It also allows subscribers to specify strategies for the permissible response time. Moreover, it continuously monitors the stream of image events while keeping track of the number of subscribers to detect objects according to keywords subscribed by subscribers. Each iteration begins with the arrival time of subscription and identification of all keywords belonging to subscription. Then for each keyword, we predict objects using our adaptation engine driven by image events, specified strategies, and properties of subscriptions. Finally, subscribers get notified based on identified objects. 
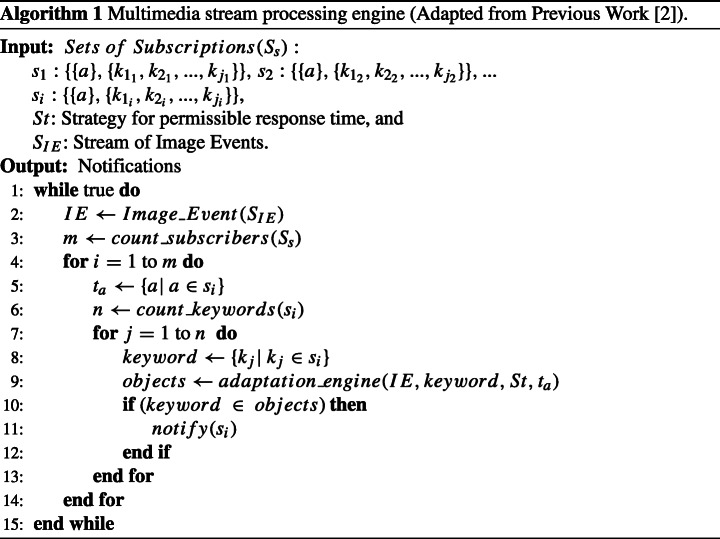

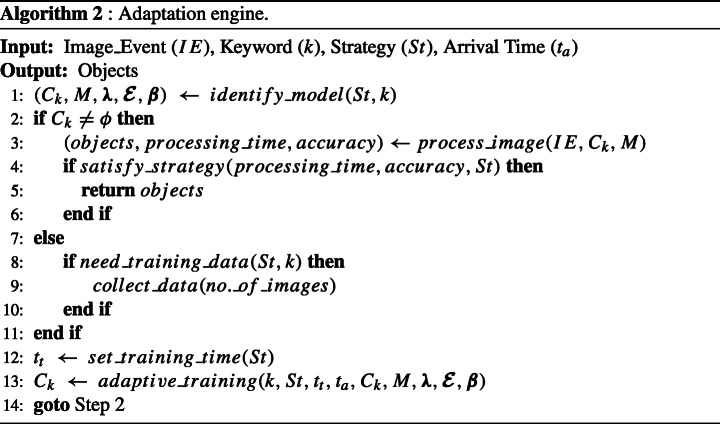


The primary role of the adaptation engine (Algorithm 2) is to identify the suitable classifier and predict objects based on specified strategy and subscribed keywords while limiting the processing time up to the permissible response time. First, it attempts to identify the suitable object detection model with specific classifiers suitable for the keyword, along with domain for hyperparameters (*λ*, $ \mathcal {E} $, *β*). In case *C*_*k*_ = *ϕ*, the procedure seeks to find the availability of training data for the keyword in existing object detection datasets present in resources of the model (please see Fig. 3). Then we use the training data to train the model for the intended classifier while setting the training time and utilising the derived parameters. Finally, after training of the classifier, we try to process image events and return objects if processing time (including training and testing), as well as accuracy, is according to the strategy. However, in the worst case, if we do not find the intended keyword-based training data in resources, we also provided the facility of collecting iconic images from the web for such unseen keywords.

Please note that the proposed model is simulated only for adaptation with hyperparameter tuning, but presented architecture is flexible to incorporate any other types of adaptation techniques in the future.

## Evaluation

This section first describes the evaluation methodologies, including details of experiment setup, evaluation metrics, and response-time focused strategies. We also show the trade-off of performance with response-time before and after adaptation, along with derived configuration parameters and the experimental results for the proposed strategies. Experiments have been conducted on Ubuntu 16.04.3 LTS (GNU/Linux 4.13.0-26-generic x86_64), with NVIDIA TITAN Xp GPU at 33MHz, whereas all object detection models use the same implementation platform Keras^6^.

### Evaluation methodology

The evaluations present in this paper divides into two categories: online classifier construction with adaptation model and without adaptation model. Firstly we analyse the trade-off between response time and performance (mAP) using default hyper-parameters (without adaptation) on object detection models YOLO, SSD, and RetinaNet [49, 51, 59]. Then we are changing the configuration with hyperparameter tuning to adapt the object detection models for low response time. We are presenting three strategies: *Minimum Response Time needed while Minimum Accuracy allowed*, *Optimal Response Time needed while Optimal Accuracy allowed*, and *Maximum Response Time allowed while Maximum Accuracy needed*, which are part of the proposed adaptation model (shown in Fig. 3). Finally, using the performance-response time trade-offs on derived hyperparameters, we identified the suitable models. Since accuracy is not only the best measure for analysing machine learning-based models, we also show the snapshots of confusion matrices for all strategies on multiple subscriptions. We utilise the Pascal VOC and Open Images datasets, to realise multimedia event streams [30, 45], where training and testing of classifiers are done on subscriptions *cat*, *dog*, *laptop*, *car*, *bus*, *bicycle*, and *football* classes with the number of training events 1804, 2204, 5528, 2820, 847, 1108, and 4339, and testing events 384, 538, 355, 1588, 256, 396, and 413 respectively. Moreover, we use the Hyperopt^7^ library with Tree-structured Parzen Estimator (TPE) [9], to derive hyperparameters in the multidimensional space of object detection models.

#### Evaluation Metrics

**Fig. 4:**
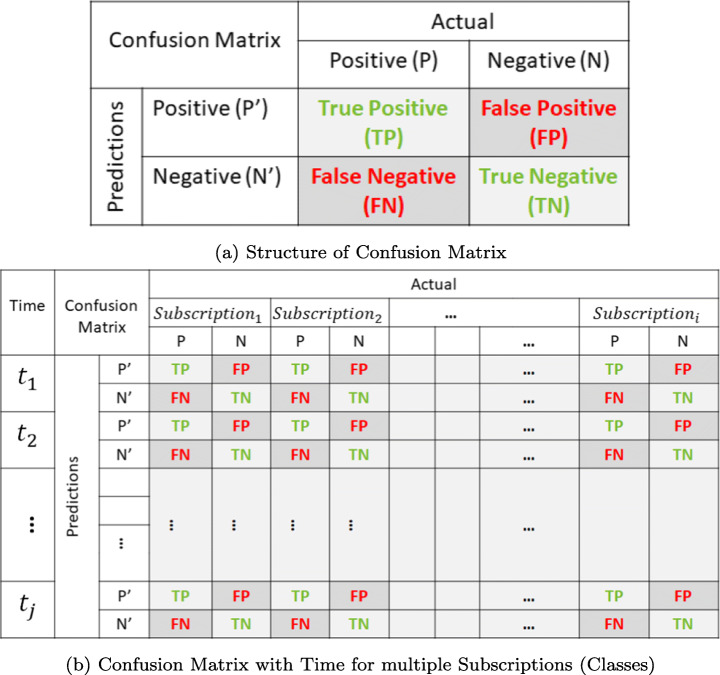
General structure confusion matrix for evaluations

- **Response Time:** It represents the time difference between the arrival (*t*_*a*_) and notification (*t*_*b*_) of subscription.
- **Accuracy:** The accuracy is the ratio of correctly predicted observation to the total observations. It is important to note that by *optimal* accuracy in this work, we mean the best accuracy that can be provided by an object detection model in a specified response-time.
- **Mean Average Precision (mAP):** The mAP is the average of the average precision of all classes. It is computed by calculating AP separately for each class, then average over them. So, the resulting mAP could be moderate, but the model might be useful for specific classes and bad for other classes. Indeed, mAP is widely considered as a good relative metric and has more agreement for the comparison of old and new methods of object detection. To verify the evaluations of mAP of the proposed adaptation model, we also present individual values of precision-recall in Appendix.
- **Confusion Matrix:** A confusion matrix contains information about actual and predicted classifications done by a classification system [58]. A confusion matrix of binary classification has four different categories: true positives, false positives, true negatives, and false negatives. The actual labels (values) form columns and predicted labels (values) form rows. The basic structure of the confusion matrix is shown in Fig. 4a. Here, TP represents the number of true positives (model predicted positive and class is also present), TN represents the number of true negatives (model predicted negative and class is also absent), FP represents the number of false positives (model predicted positive, but class is absent), FN represents the number of false negatives (model predicted negative, but class is present). We show the confusion matrix for multiple subscriptions at a regular interval of time in our evaluations, to show the exact number of actual and predicted subscriptions. Figure 4b represents its general structure.

#### Strategies

Based on accuracy-response time trade-off characteristics, the requirements of high-performance execution method (presently object detection methods) can be achieved using the following three main strategies:

1. **Minimum Response Time needed while Minimum Accuracy allowed:** The strategy “Minimum Response Time needed while Minimum Accuracy allowed” includes the computation of accuracy that we can achieve by setting limits to response time until it reaches a certain threshold, which is 15 min (including both training and testing time) in the present work by considering requirements of real-time systems.
2. **Optimal Response Time needed while Optimal Accuracy allowed:** Similarly, this strategy “Optimal Response Time needed while Optimal Accuracy allowed” focuses on achieving the optimal accuracy while allowing response time of few hours (1 hour in the present case) for the training and testing of neural network-based object detection models.
3. **Maximum Response Time allowed while Maximum Accuracy needed:** The “Maximum Response Time allowed while Maximum Accuracy needed” would be able to cover existing scenarios of object detection models where models are allowed to train for the extended number of hours to achieve the maximum accuracy. Since this strategy focuses only on maximizing accuracy, it results in high response time, and thus not feasible for real-time scenarios.

In addition to the strategies considered here, we may also design more strategies in the future based on a higher rate of change, approximately-zero-response time, and constant-accuracy. We have conducted experiments using only the above three strategies, since these are highly distinguishable among themselves in terms of response-time, in analysing the best performance on the detection of multimedia events.

### Online classifier construction before adaptation

#### Response time vs performance

**Fig. 5:**
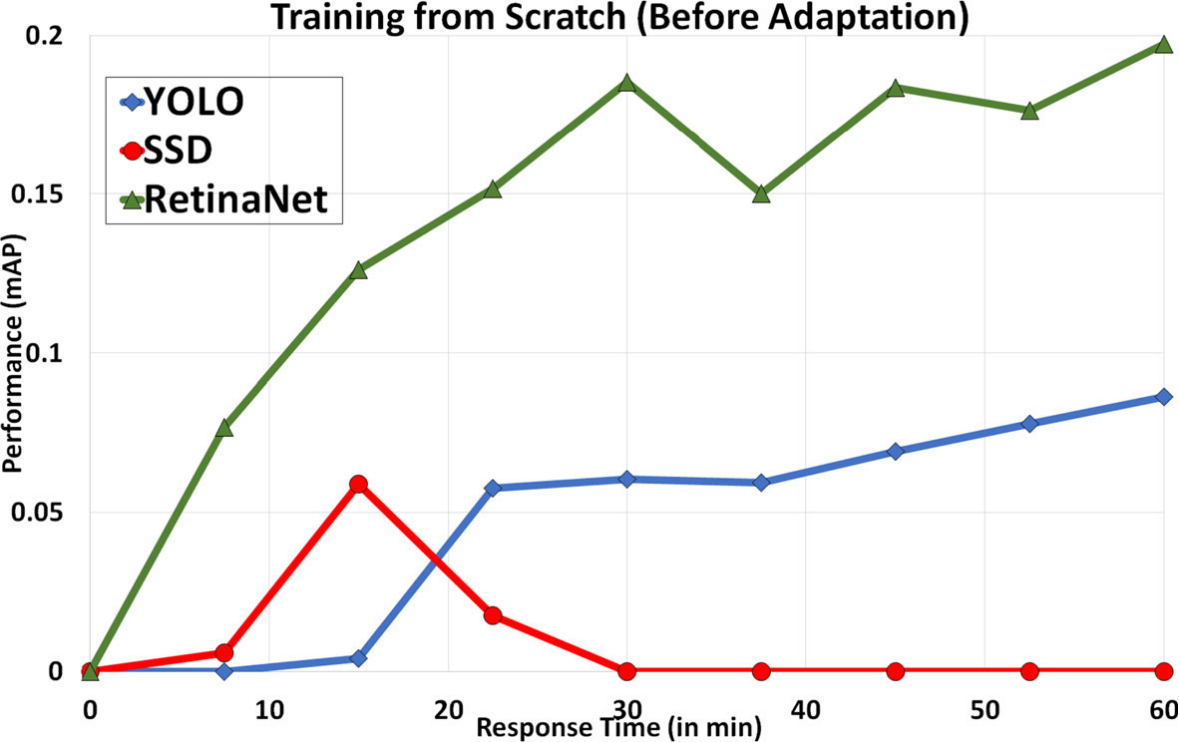
Performance vs Response time without adaptation (for 15-min and 60-min intervals)

Figure 5 represents the performance of the proposed model with response time while training from scratch on the arrival of a new subscription. We observe that all three object detection models (YOLO, SSD, RetinaNet) provide low values with the mean average precision (mAP) in low response-time. The maximum performance for the SSD model reaches up to 0.06, YOLO accomplishes mAP 0.09, and RetinaNet achieves mAP 0.20. Among these different models, SSD performs average at 15 *min* of response-time and worse in 1 *hour* of response-time. However, YOLO performs average in 1 *hour* but not in 15 *min*. RetinaNet provides better than both YOLO and SSD models while having mAP of 0.13 in 15 *min* and 0.20 in 1 *hour* for the training from scratch for new subscriptions. We can also note the SSD performance is increased initially and then decreased. This also validates the key difference presented in the SSD model [51], that SSD does not make random guesses like other detectors at the start of the training process, but it assigns ground truth boundary boxes to default boxes. Although 15 min and 1 hour is very less time for the training of classifiers (that require up to days), hence training can be very unstable at early stages for any detector.

Default hyperparameters suggested by object detection models that we used for analysing the performance and time trade-off are present in Table 3 with their respective accuracy achieved on new subscriptions while using different strategies. The derived accuracies for both strategies S1 (Minimum Response Time needed while Minimum Accuracy allowed) and S2 (Optimal Response Time needed while Optimal Accuracy allowed) state that none of these models are applicable before adaptation and necessitates further investigation after adaptation. Moreover, we can easily conclude that all models are equally suitable only for strategy S3 (Maximum Response Time allowed while Maximum Accuracy needed) at their default configuration, due to having low accuracies on reduced response timings. Please note all models train from scratch without the use of any pre-trained model. Although for the maximum response time of “S3”, we are using fully trained weight files provided by object detection models along with their respective recommended backbones *darknet*, *VGG16*, and *resnet50* [49, 51, 60].

**Table 3:** Default hyperparameters with accuracy for different strategies

	Default Hyperparameters			Accuracy with Response Times			
Object Detection Models	Batch Size	Learning Rate	#Epochs	S1: Min Res & Min Acc Time	S2: Opt Res & Opt Acc Time	S3: Max Res & Max Acc Time	Frame Rate
			3	0.00%			
YOLOv3	64	0.001	10		79.16%		114fps
			$_{\sim }$300			98.53%	
			1	10.08%			
SSD300	32	0.001	3		54.79%		21fps
			$_{\sim }$120			98.58%	
			4	64.66%			
RetinaNet	1	1e-5	14		74.87%		7fps
			$_{\sim }$50			98.62%	

Other than accuracy with response time, we are also reporting frame-rate of the object detection models, which are useful to determine the best model if we have trained classifiers available. On arrival of any “unknown” subscription, the first response time could be 15-min or 1-hour because of the training of the classifier. That subscription (keyword) will become “known”, and the next response time will depend only on the testing time, *i.e.* frame-rate of object detection model. Presently the predicted response-time of the proposed model for *known* subscriptions using YOLOv3, SSD300, and RetinaNet are 0.009, 0.05, and 0.08 seconds respectively.

#### Results for proposed strategies

Further, experiments have been conducted for strategy S3 using defaults hyperparameter configurations suggested in Table 3 on multiple subscriptions. Confusion matrix has been shown by taking SSD as an object detection model presently (can be changed to RetinaNet or YOLO) in Table 9, where it contains information about expected and predicted classes detected by the proposed system. Here strategy S3 could serve as an oracle, and its prediction counts show the maximum performance that we could achieve. We can observe that the values of true positives and true negatives are considerably higher than the values of false positives and false negatives for most of the subscriptions (please see Fig. 4 for the details of the confusion matrix). Hence, S3 gives the upper bound of TP and TN, as well as lower bounds of FP and FN. This also concludes that if we allow our model to get trained for the maximum amount of time (up to days), then our model will be able to achieve much higher accuracy ($\sim 98.58\%$) even for any previously “unknown” subscription.

However, if a user wants to reduce the first response time, we need to move towards the adaptation of object detection models. Our model achieves this by facilitating strategies S1 and S2 for users and hyperparameter tuning for the adaptation.

### Online classifier construction after adaptation

#### Hyperparameter tuning

**Fig. 6:**
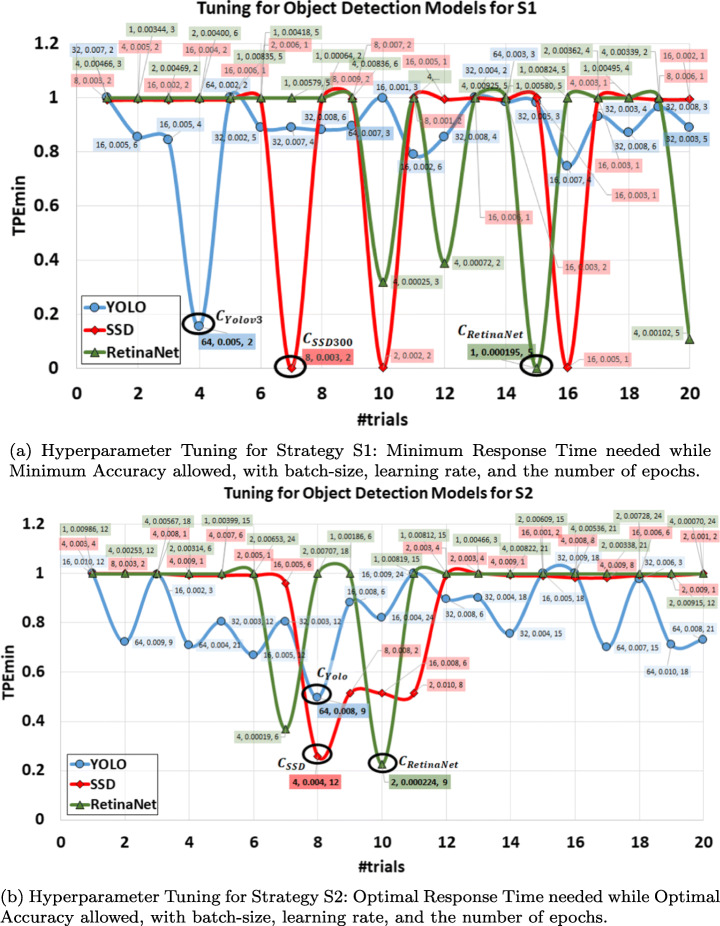
Hyperparameter tuning for 15-min and 1-hour training

Hyperparameter tuning is utilized for the self-optimization of the model on the requested strategies. The goal of tuning is to find the best values of hyperparameters in a given space using a specific function. It mainly requires the objective function to minimise, the space to search hyperparameters, and the method of searching, to output the point of evaluations. Figure 6 represents hyperparameter tuning of object detection models by considering 20 number of trials and the TPE search method [9], which need to be minimized based on mean average precision (mAP). The search space that we used to tune the hyperparameters batch-size (*β*), learning rate (*λ*), and the number of epochs ($ \mathcal {E} $), is shown in Table 4. It is important to note that we chose the domain space of hyperparameters according to the limitation of our resources, and thus could change in the future.

**Table 4:** Space defined for hyperparameter tuning for the scratch training of 15-min and 1-hour

Object Detection Models	15-min Training			1-hour Training		
	Batch Size	Learning Rate	#Epochs	Batch Size	Learning Rate	#Epochs
YOLOv3	{1, 2, 4, 8, 16, 32, 64}	[0.001, 0.1]	[1,6]	{1, 2, 4, 8, 16, 32, 64}	[0.001, 0.1]	[1,24]
SSD300	{1, 2, 4, 8, 16}	[0.001, 0.1]	[1,2]	{1, 2, 4, 8, 16}	[0.001, 0.1]	[1,12]
RetinaNet	{1, 2, 4}	[0.00001, 0.01]	[1, 6]	{1, 2, 4}	[0.00001, 0.01]	[1, 24]

Tuning for the strategy S1 “Minimum Response Time needed while Minimum Accuracy allowed” with specific values of *β*, *λ*, and $ \mathcal {E} $, for each trial, are shown in Fig. 6a. This attempts to find the parameters that may give the highest performance within 15 min of training for any “unknown” subscriptions. As the full training time of each object detection model is up to days, it is hard to train a model within only 15 min (or even in 1 hour). Thus, no model indicates any accuracy for most of the combinations of hyperparameters and shows the maximum value for TPE (which is based on the inverse of the mAP). However, we find a few combinations of hyperparameters that give average accuracy even within the 15 minutes of training time, and that shows the sudden minimum for those few values. We observe in the case of YOLO the model is reaching a minimum TPE for the largest batch-size of 64. Moreover, it also requires a higher learning rate (0.005) close to the highest value (0.008) in the case of YOLO. Although the number of epochs found is 2 for the minimum TPE and the highest value of the number of epochs that we could achieve in 15 min is 6.

We found that the SSD model is slowest in training and cannot train more than 2 epochs in 15 min. In this case, we get the minima at three points: (8, 0.003, 2), (2, 0.002, 2), and (16, 0.005, 1) which proves that even with the lower number of epochs we can achieve average accuracy by altering the batch-size and keeping high learning rates. We choose *β* = 8, *λ* = 0.003, and $ \mathcal {E}=2$ for SSD in our experiments, which could switch to any other two data points. RetinaNet model achieves its minimum value at data point (1, 0.000195,5), which represents the lowest learning rate as well as the lowest batch size among all trials. However, the RetinaNet model reaches up to 5 epochs with such low learning rates within 15 min of training.

Similarly, tuning for finding the best parameters for strategy S2 “Optimal Response Time needed while Optimal Accuracy allowed” is shown in Fig. 6b. Here we found the minimum value of TPE function for YOLO model at data point (64, 0.008, 9) which again (same as S1) we found at the highest value of batch-size, and high learning rate, while having a low number of epochs (9) as the highest value achieved could be 21. The SSD model found its minima at (4, 004, 12), which indicates the highest number of epochs in 1-hour training. RetinaNet for S2 follows the same trend as S1 and found its minimum point at the lowest learning rate (0.000224), low batch-size (2), and epoch value reached till 9 where possible highest value of epoch could be 24.

We use the derived data points to investigate the maximum performance that we can achieve using different models while analysing the trade-off of performance with response time for strategies S1 and S2.

#### Response time vs performance

**Fig. 7:**
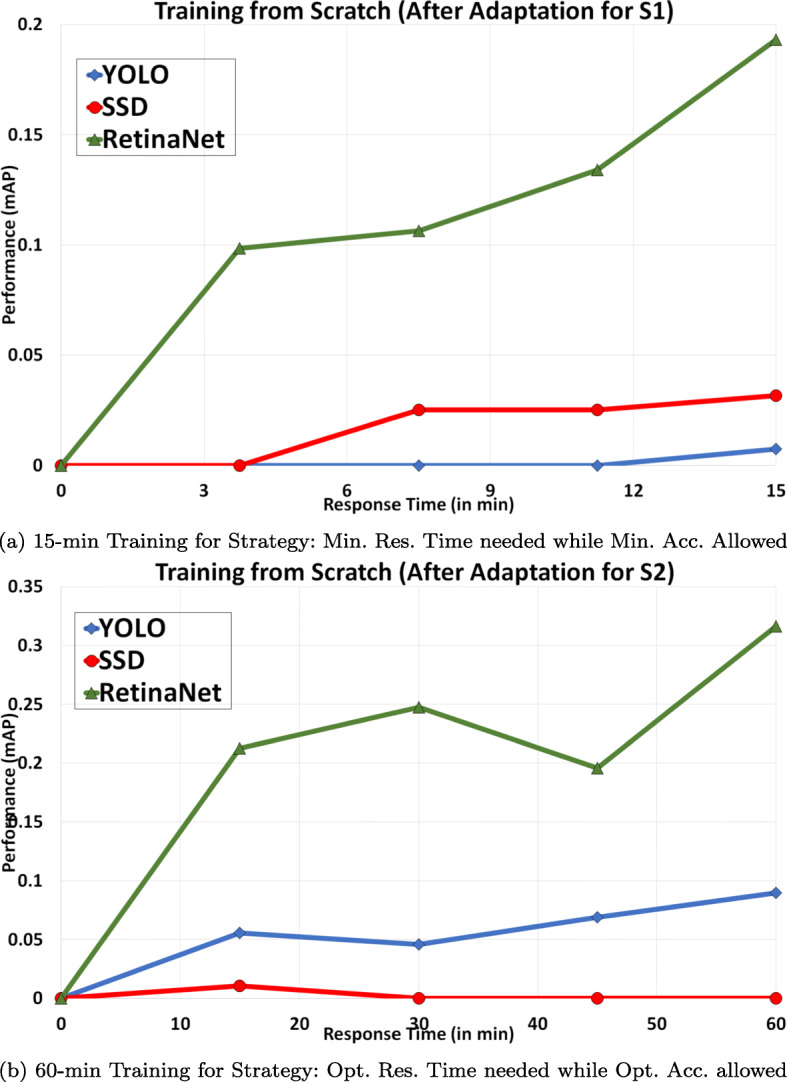
Performance vs Response time after adaptation (for 15-min and 60-min intervals)

Figure 7 represents a trade-off of performance (mAP) with response time after adaptation (tuning hyperparameters) of the proposed model for the processing of new subscriptions with strategies S1 and S2. The performance of the proposed multimedia event detection model has been evaluated on the best configuration hyperparameters (*learning rate*, *batch size*, and the *number of epochs*) derived in previous Section 5.3.1, for the training of 15 min and 1 hour. We observe that the RetinaNet model is performing better than YOLO and SSD for strategy S1 (please see Fig.7a). Moreover, its performance is also enhanced to the precision of 0.20 (after adaptation) from 0.13 (before adaptation). Similarly, Fig. 7b shows the mAP for strategy S2 with a response-time of 1-hour. Here, also RetinaNet outperforms, and its precision increased from 0.20 (before adaptation) to 0.32 (after adaptation).

Results of achieved accuracies after adaptation along with derived hyperparameters of object detection models for both strategies S1 and S2 are shown in Tables 5 and 6. We found that the accuracy of each model before adaptation (Table 3) increases after adaptation (Table 5), for strategy S1. Specifically it increases from 0.00% to 5.66%, 10.08% to 47.32%, and 64.66% to 79.00% for YOLO, SSD, and RetinaNet respectively. Correspondingly, we also get better accuracy for strategy S2 after adaptation (Table 6) than before adaptation (Table 5). YOLO increased from 79.16% to 82.82%, SSD slightly changed from 54.79% to 54.81%, and RetinaNet considerably increased from 74.87% to 84.28%.

**Table 5:** Derived hyperparameters with accuracy for strategy-1

Object Detection	Computed Hyperparameters			Accuracy for S1: Min. Res. &
Models				Min. Oct. Time
	Batch Size	Learning Rate	#Epochs	
YOLOv3	64	0.005315	2	5.66%
SSD300	8	0.002612	2	47.32%
RetinaNet	1	0.000195	5	79.00%

**Table 6:** Derived hyperparameters with accuracy for strategy-2

Object Detection	Computed Hyperparameters			Accuracy for S2: Opt. Res. &
Models				Opt. Acc. Time
	Batch Size	Learning Rate	#Epochs	
YOLOv3	64	0.007935	9	82.82%
SSD300	4	0.003600	12	54.81%
RetinaNet	2	0.000224	9	84.28%

We conclude that RetinaNet is performing best among all object detection models on such low training times. Thus, we can easily consider RetinaNet with its derived configuration for the detection of objects for both strategies S1 and S2. Since recall is also a popular evaluation metric but not considered useful for the comparison of object detection models, we present an analysis of precision-recall with the change in response-time in Appendix. Other than communicating the change in values of recall with response-time, these precision-recall curves clearly show that the Area Under Curve (AUC) is relatively bigger after adaptation than before adaptation. Higher values for time-based AUC for RetinaNet also supports its high precision and recall for both 15 min and 1-hour training.

#### Results for proposed strategies

Table 7 represents the results of the proposed adaptation model for Strategy-1 “Minimum Accuracy and Minimum Response Time” using RetinaNet as an object detection model with 15-min of training from scratch. Similarly, Table 8 represents the results for Strategy-2 “Optimal Accuracy and Optimal Response Time” until one hour. Here values of true positives (TP) and true negatives (TN) shown in light colour should increase with time and values of false positives (FP), and false negatives (FN) shown in dark colour should decrease with time.

We observe that even within 15 min of training, we get remarkable counts for TP and TN, following high values of FP and FN. Table 7 shows that TPs are increasing extensively in four cases and decreasing in three cases with time. However, TNs are increasing in three cases, and in four cases, its count is decreasing. Similarly, FP and FN are decreasing considerably in three cases, but not in four cases. So, we conclude that the model is not stable in 15 min of training and requires more time to train completely. Despite that, it provides an average accuracy of 79.00% (using RetinaNet) within 15 min, and we could consider it suitable in situations where we need a quick response and compromise in the accuracy is allowed.

Additionally, when we apply the derived hyperparameters for the strategy S2, TP values increase for most of the classes within 1-hour of training as compared to values at 15 min of training. Here, values of TNs are increasing and decreasing with time, and values of FPs are decreasing and increasing as well. Although FNs are decreasing in the majority of cases for 1 hour of training. The average accuracy computed from the confusion matrix (shown in Table 8) is 84.28% for S2 using RetinaNet.

It is worth noting that the total number of input images at different time intervals is the same in each subscription, and the number of instances detected in an image could be different for distinct models. For instance, if we give an input image consisting of two cats, and our model after 7 min of training detects five cats, then we will have *TP* = 2, *FP* = 3, *TN* = 0, and *FN* = 0 (*i.e.total* *number* *of* *instances* = 5 at 7 min). On the other hand, if our model after 15 min of training detects three cats, then we will have *TP* = 2, *FP* = 1, *TN* = 0, and *FN* = 0 (i.e. *total* *number* *of* *instances* = 3 for 15 min). These multiple detections in an image make the resulting total number of instances different in the confusion matrix of *object detection*, unlike the case of conventional *image classification*, where an image could either just “belong” or “not belongs” to a particular class. Nevertheless, there is still a gap between the values (TP, FP, TN, and FN) for strategies (S1 and S2) and oracle strategy S3 (Table 9 discussed in Section–5.2.2), which is explicit because of their large gap in response-time.

## Conclusion and future work

In this paper, we proposed an adaptive approach for multimedia event processing using online classifier construction of object detection models for the handling of unknown subscriptions with a low response-time. The proposed model is optimised with the tuning of hyperparameters of existing object detection models YOLOv3, SSD300, and RetinaNet. Experiments demonstrate that the trade-off between performance and training time with adaptation could be useful to reduce the overall response time by compromising the accuracy. The proposed system achieves an accuracy of 79.00% with 15 min training and 84.28% with 1-hour of training on a single GPU, which is reasonable for the detection of objects for unknown subscriptions on such low training times. In future work, it can be useful to analyse the system with additional object detection models, and with the inclusion of more response-time based strategies. We can also extend the proposed model for the overlapping scenarios or further optimize for related domain subscriptions.

## Figures and Tables

**Fig. 8:**
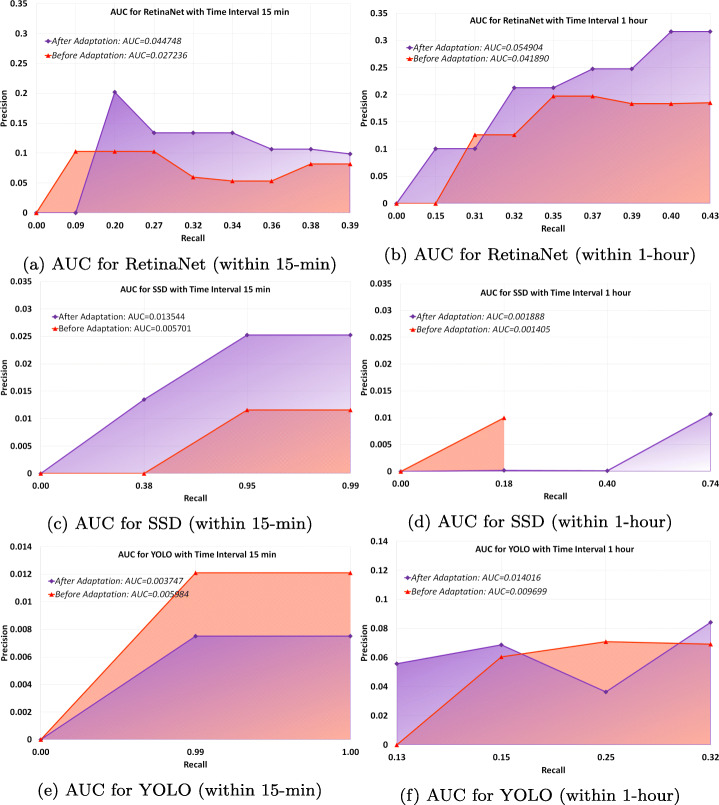
Area Under Curve (AUC) before and after proposed adaptation within response-time interval of 15 min and 1 hour using different object detection models

## Appendix: Response-time driven precision-recall area under curve (AUC)

The precision-recall curves visualise the performance of classification models while summarizing the trade-off between precision and recall using a range of thresholds. A high area under the curve represents high scores for both precision and recall, which shows that the classifier is returning accurate results (high precision) and the majority of all positive results (high recall). Area Under Curve (AUC) is an approximation of the area under the precision-recall curve [13]. AUC is desirable to evaluate which model is performing better and what should be the value of the threshold to achieve maximum precision as well as recall. However, in our case we already have values of threshold evaluated by different object detection models (YOLO:0.25, SSD:0.45, RetinaNet:0.50). Moreover, we need to evaluate these models before and after the proposed adaptation within the short interval of response-time. Thus, we show the precision-recall curves by plotting all data points of precision and recall computed within the response-time of 15 min and 1 hour in Fig. 8, and verify that values of AUC *after adaptation* are relatively bigger (higher) than *before adaptation*.

**Table 7:** Confusion matrix for strategy S1: minimum response time needed while minimum accuracy allowed

Time	Matrix		Subscriptions (Expected)													
			Cat		Dog		Laptop		Car		Bus		Bicycle		Football	
			P	N	P	N	P	N	P	N	P	N	P	N	P	N
7 min	Predictions	P’	185	3050	24	652	37	160	112	230	183	4969	178	2142	0	0
		N’	180	4768	506	4928	162	365	1429	4846	71	4790	211	4812	220	401
15 min		P’	0	2	301	7489	64	201	219	281	2	19	37	57	82	27
		N’	370	4952	229	4674	135	339	1322	4749	252	4950	352	4918	138	321

**Table 8:** Confusion matrix for strategy S2: optimal response time needed while optimal accuracy allowed

Time	Matrix		Subscriptions (Expected)													
			Cat		Dog		Laptop		Car		Bus		Bicycle		Football	
			P	N	P	N	P	N	P	N	P	N	P	N	P	N
15 min	Predictions	P’	322	7650	13	218	52	47	246	150	110	1278	52	143	69	13
		N’	48	4638	517	4939	147	349	1295	4721	144	4851	337	4915	151	335
30 min		P’	166	2058	10	68	135	373	144	36	222	8477	163	615	150	110
		N’	204	4786	520	4942	64	275	1397	4823	32	4780	226	4822	70	257
45 min		P’	160	1444	13	78	58	27	628	555	181	2038	228	1616	117	24
		N’	210	4793	517	4939	141	343	913	4475	73	4800	161	4771	103	286
60 min		P’	117	724	126	575	127	93	680	555	173	950	133	105	116	25
		N’	253	4835	404	4828	72	278	861	4441	81	4801	256	4836	104	288

**Table 9:** Confusion matrix for strategy S3: maximum response time allowed while maximum accuracy needed

Time	Matrix		Subscriptions (Expected)													
			Cat		Dog		Laptop		Car		Bus		Bicycle		Football	
			P	N	P	N	P	N	P	N	P	N	P	N	P	N
Upto	Predi-	P’	318	52	438	69	97	20	1018	523	180	74	280	109	15	7
Days	ctions	N’	19	4610	92	4475	102	224	79	4141	20	4754	23	4684	205	190

The performance of RetinaNet before and after adaptation shown in Fig. 8a and b. It can be seen the precision-recall curve covers more area after adaptation in both cases of 15 min and 1-hour response-time, thus also have relatively better values for AUC as compared to the actual AUC of RetinaNet model (i.e. without/before adaptation) within such short training time. Analysis of the SSD object detection model (Fig. 8c and d) shows its lower performance but also verifies the improvement in performance after adaptation than before. AUC for YOLO after adaptation is better than before adaptation for response-time of 1 hour (Fig. 8f). Here, the performance of YOLO for the 15 min training time get decreased after adaptation, which could be the reason YOLO is still struggling with accuracy and not considered very reliable as compared to other object detection models [72, 76]. However, other than the AUC of YOLO for 15 min response-time, we can conclude that the proposed adaptation strategy is effective in all cases using all object detection models (Fig. 8).

It also verifies that the RetinaNet model with adaptation performs the best, for both cases of 15 min and 1 hour response time. Thus RetinaNet with its derived configuration is suitable for both Strategies 1 and 2 shown in Tables 5 and 6. Specifically, the peak values found for precision and recall within a time interval of 15 min are 0.20 and 0.20, respectively. Lastly, the highest values of precision and recall are 0.32 and 0.43, respectively, for the response time of 1 hour.

## Footnotes

1 https://github.com/EscVM/OIDv4_ToolKit

2 https://github.com/tzutalin/ImageNet_Utils

3 https://github.com/funpokes/bing-image-search

4 https://github.com/hardikvasa/google-images-download

5 https://www.flickr.com/services/api/

6 https://keras.io/

7 https://github.com/hyperopt/hyperopt
